# The Effect of Hydroalcoholic Extracts of Medicinal Plants on Fungi Isolated From Toilet and Nursery Surfaces in a Daycare Center: An In Vitro Study

**DOI:** 10.7759/cureus.34013

**Published:** 2023-01-20

**Authors:** Elena Carla Batista Mendes, Dora Inés Kozusny-Andreani, Rogério Rodrigo Ramos, José Martins Pinto Neto, Luciana Estevam Simonato, André Wilian Lozano, Wagner Rafael da Silva, Luis Lenin Vicente Pereira, Daniela da Silva Garcia Regino, Carla Maria Zordan Geraldo de Moraes

**Affiliations:** 1 Department of Health Sciences, University Center of Santa Fé do Sul, Santa Fé do Sul, BRA; 2 Department of Health Sciences, Brasil University, Fernandópolis, BRA; 3 Department of Research, Faculty of Roseira, Roseira, BRA

**Keywords:** palliative care, medicinal plants, infectious diseases, dermatophytes, candida albicans, fusarium spp, aspergillus niger, natural antifungals, environmental health

## Abstract

Background

Brazil has the most extensive plant genetic biodiversity in the world. Knowledge regarding the therapeutic properties of medicinal plants obtained through popular medicine has been accrued over centuries. Such empirical knowledge often symbolizes the only therapeutic resource for various ethnic communities and groups. This study aimed to evaluate the efficacy of hydroalcoholic extracts of medicinal plants in controlling isolated fungi found in bathrooms and nurseries of a daycare center in the northwestern region of São Paulo state.

Methodology

This is an in vitro study carried out in the microbiology laboratory. The analyzed fungi were *Aspergillus niger*, *Fusarium* spp., *Trichophyton mentagrophytes*, *Microsporum gypseum*, and *Candida albicans*. These fungi were exposed to the hydroalcoholic extracts of rosemary, citronella, rue, neem, and lemon.

Results

Rue extract was more effective against *Candida albicans* at a concentration of 12.5%. Citronella was effective against *Aspergillus niger* and *Trichophyton mentagrophytes* at a concentration of 6.25%. Lemon was effective against *Fusarium* spp. at a concentration of 6.25%.

Conclusions

The hydroalcoholic extracts showed antifungal activity. The in vitro evaluation of medicinal plants showed that the extracts of rue, citronella, and lemon showed a fungicide effect.

## Introduction

Many medicinal plants have been acknowledged as a valuable resource of natural antimicrobial compounds and an alternative that can effectively treat bacterial and fungal infections [1]. Globally, there is vast plant genetic diversity. Knowledge regarding the therapeutic properties of medicinal plants has been accumulated for centuries from popular medicine. Such empirical knowledge is often the only therapeutic resource for several communities and ethnic groups. This information, combined with current scientific studies, has contributed significantly toward understanding the medicinal properties of plants [2-9].

Medicinal plants are an essential therapeutic resource in treating diseases, from which the source material for manufacturing herbal medicines is extracted. Many plant species have pharmacological properties such as glycosides, saponins, flavonoids, steroids, tannins, alkaloids, and terpenes. They contain various secondary metabolites, which can be used to combat disease-causing pathogens [9-13].

The antimicrobial properties of plant extracts and essential oils have been scientifically proven, and these properties have driven many researchers to study biological plant activities, given their widespread use. These studies have been boosted due to the increase in microorganisms resistant to most known antimicrobials [1,14]. Using different plant parts, such as leaves, fruits, roots, and bark, can be a sustainable, viable, and affordable alternative for treating microorganisms [7,15-17].

Medicinal plants can be used to increase the technological resources in the disinfectant and antiseptic fields, avoiding possible adverse effects that some synthetic chemicals may have on the patient, host, environment, and causal agent resistance, as well as reducing hygiene costs [1,18,19].

Therefore, this study aimed to evaluate the effect of hydroalcoholic extracts obtained from medicinal plants on isolated fungi found on the toilet and nursery surfaces in a daycare center.

## Materials and methods

Preparation of medicinal plant extracts

Leaves of rue (*Ruta graveolens* L.), rosemary (*Rosmarinus officinalis* L.), citronella (*Cymbopogon winterianus* Jowitt), neem (*Azadirachta indica* A. Juss), and lemon (*Citrus limon* (L.) Burm. f.) were used. The plants were identified by a specialist from the faculty of medicine. The methodology described by Pereira et al. was applied to obtain the extracts with a few modifications [20]. The leaves were rinsed using water, and, subsequently, the raw material was dried in a kiln at 33°C for one week to dehydrate plants and stabilize the enzyme content. Then, the raw material was removed from the kiln, crushed into a powder in an electric mill, and submitted to the active principles extraction process. The extraction method was leaching or percolation in a continuous flow at room temperature.

Due to the material being rich in polyphenols amenable to easy structural modification, hot extraction was not used to preserve the stability of the material. In the continuous flow leaching, a constant refill of the extracting solution (80% hydroalcoholic solution) for 24 hours was applied. Once this interval had elapsed, complete extraction of the markers or active ingredients was performed. In this phase, a ratio of 8 L of hydroalcoholic solution for 1 kg of dry and pulverized raw material was used for the complete depletion of the active principles. It recovered a volume of approximately 500 mL of each extract. After filtration to remove impurities, it was placed in clean and dry amber flasks and stored at 5°C.

Fungal strains and growth medium

Strains of *Aspergillus niger* (*A. niger*), *Fusarium* spp., *Candida albicans* (*C. albicans*), *Microsporum gypseum* (*M. gypseum*), and *Trichophyton* *mentagrophytes* (*T. mentagrophytes*) isolated from toilet and nursery surfaces of a daycare center were used [21].

*M. gypseum* and *T. mentagrophytes* strains were reactivated in Sabouraud-Dextrose agar medium (SDA, Oxoid®) with chloramphenicol and incubated for seven days at 35°C, while *C. albicans* was cultivated in SDA at 35°C for 24 hours. To cultivate *A. niger* and *Fusarium* spp. we used potato-dextrose agar medium (PDA, Kasvi®) and incubated for five days at 28°C.

Filamentous fungi were cultivated in Sabouraud broth (SDB, Oxoid®) for inoculum development to determine the minimum inhibitory concentration (MIC) and kept with chloramphenicol under an orbital shaker (200 rotations per minute (rpm)) for five days at 35°C, while *C. albicans* were inoculated in SDB, incubated for 24 hours, and kept under an orbital shaker (200 rpm).

Determination of minimum inhibitory concentration and minimum fungicidal concentration

The method described by Guimarães et al. was employed to determine the MIC and minimum fungicidal concentration (MFC) of hydroalcoholic extracts [22]. The microdilution plate technique was used to determine the MIC, following the methodology recommended by the National Committee for Clinical Laboratory Standards [23,24]. The MIC was considered the lowest extract concentration capable of inhibiting fungal development.

For the MFC, 100 µL of the solution was taken from the plate wells used in the MIC and transferred to Petri dishes containing SDA and PDA. Then, the plates were incubated at 37°C for 24 hours for *C. albicans* and five to seven days for filamentous fungi. The study evaluated the growth of the cultures, and the plates presenting a fungal absence growth were assessed to determine the MFC.

Data analysis

Descriptive analysis of microbial count was done according to the different concentrations of different hydroalcoholic extracts evaluated. The study analyzed microbial count data through line graphs to observe the microbial count progression as the essential oil concentration increased. The Kruskal-Wallis [25] test was applied to compare the microbial count variation relative to the concentration and analysis time, with the latter referring to the MFC. Minitab 15® (Minitab, Inc., State College, PA, USA) and InStat® were used for data analysis.

## Results

Table 1 shows the MIC and MFC values for the different plant extracts in controlling fungal species.

**Table 1:** Minimum inhibitory and fungicidal concentrations of hydroalcoholic extracts of medicinal plants on different fungal species. %: hydroalcoholic extracts concentration in percentage.

Extracts	Microorganisms				
	Candida albicans	Aspergillus niger	Microsporum gypseum	Trichophyton mentagrophytes	*Fusarium *spp.
Minimum inhibitory concentration					
Rosemary	25%	50%	50%	50%	12.5%
Lemon	50%	25%	50%	100%	6.25%
Neem	25%	100%	100%	50%	12.5%
Rue	12,5%	50%	50%	50%	50%
Citronella	50%	6.25%	50%	6.25%	50%
Minimum fungicidal concentration					
Rosemary	25%	50%	50%	50%	25%
Lemon	50%	25%	50%	100%	12.5%
Neem	50%	100%	100%	50%	12.5%
Rue	25%	50%	50%	50%	50%
Citronella	50%	12.5%	50%	12.5%	50%

The results revealed that rue extract inhibited the growth of *C. albicans* and *M. gypseum*. In contrast, citronella extract was effective against *A. niger* and *T. mentagrophytes*, lemon was more effective against *Fusarium* spp., and neem extract inhibited the growth of *C. albicans* and *Fusarium* spp.

**Figure 1:**
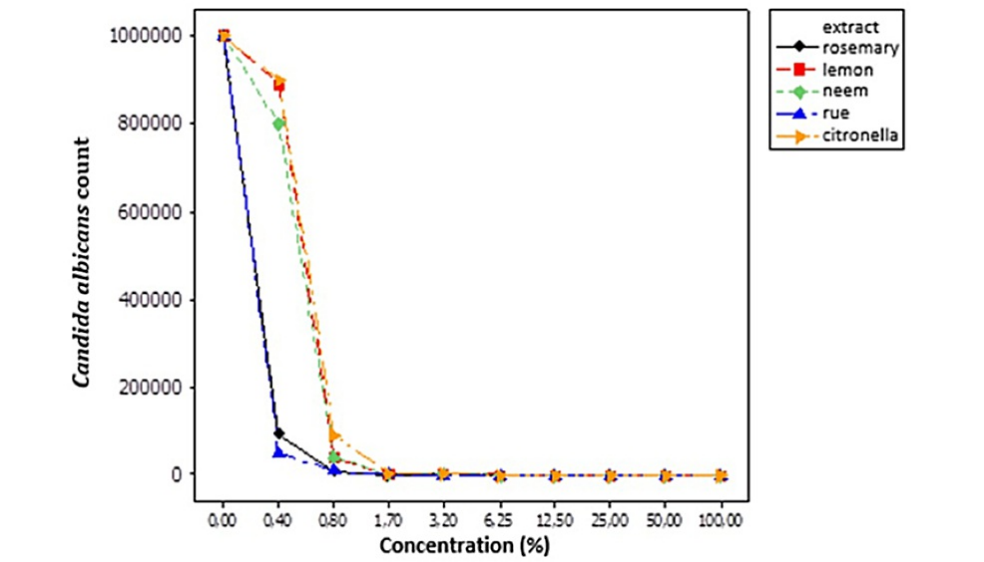
The behavior of Candida albicans in response to different hydroalcoholic extract concentrations of medicinal plants. %: numbers represent counts.

Table 2 and Figure 1 show the effects of different extracts on *C. albicans*. The study observed that the extracts reduced *C. albicans* gradually, in accordance with the concentrations used. Rue extract was efficient at the lowest concentration of 12.5%, rosemary and neem were effective at 25%, while citronella and lemon were effective at 50%.

**Table 2:** Antimicrobial activity of hydroalcoholic extracts of medicinal plants in the Candida albicans in vitro control. %: numbers represent counts; *: significant findings at p < 0.05; **: similar letters in the same column do not differ from each other at a 5% probability level (Kruskal-Wallis test); a, b: differ from each other; a, ab, b: do not differ from each other.

Concentration (%)	Extracts				
	Rosemary	Rue	Citronella	Lemon	Neem
0.0	1.1×10^6 a**^	1.0×10^6 a^	1.0×10^6 a^	1.0×10^6 a^	1.0×10^6 a^
0.4	9.5×10^4 ab^	5.0×10^4 ab^	9.0×10^5 ab^	8.9×10^5 ab^	8.0×10^5 ab^
0.8	9.2×10^3 ab^	9.0×10^3 ab^	9.0×10^4 ab^	3.9×10^4 ab^	4.0×10^4 ab^
1.7	1.0×10^2 ab^	2.0×10^2 ab^	3.2×10^3 ab^	1.2×10^3 ab^	2.0×10^2 ab^
3.2	9.1×10^2 ab^	8.0×10^1 ab^	6.5×10^2 ab^	8.0×10^2 ab^	5.5×10^2 ab^
6.25	1.5×10^2 ab^	0.5×10^1 ab^	1.0×10^1 ab^	1.0×10^2 ab^	5.0×10^1 ab^
12.5	0.4×10^1 ab^	0.0^ b^	4.9×10^1 ab^	4.7×10^1 ab^	0.6×10^1 ab^
25	0.0^ b^	0.0 ^b^	0.1×10^1 ab^	0.1×10^1 ab^	0.0 ^b^
50	0.0 ^b^	0.0 ^b^	0.0 ^b^	0.0 ^b^	0.0 ^b^
100	0.0 ^b^	0.0 ^b^	0.0 ^b^	0.0 ^b^	0.0 ^b^
P-value*	0.001	0.001	0.001	0.001	0.001

**Figure 2:**
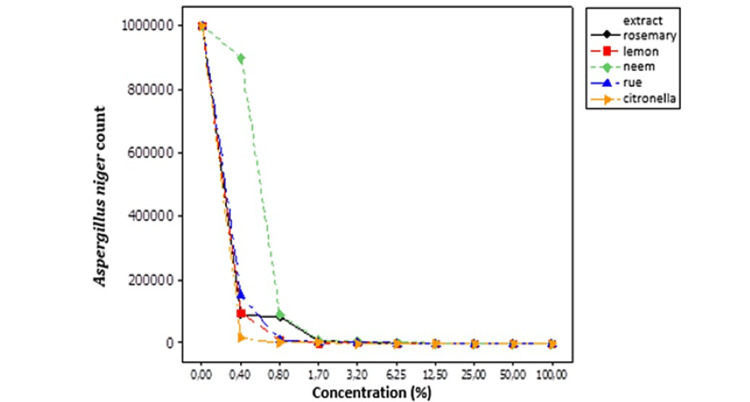
The behavior of Aspergillus niger in response to different hydroalcoholic extract concentrations of medicinal plants. %: Numbers represent counts

The control results using medicinal plant extracts to prevent *A. niger* are shown in Table 3 and Figure 2. Rosemary and neem extracts were not very effective, requiring 50% and 100% concentrations, respectively, which are considered high values. The rue and lemon extracts controlled *A. niger* when 25% concentrations were used, while citronella was more effective (12%).

**Table 3:** Antimicrobial activity of hydroalcoholic extracts of medicinal plants in the Aspergillus niger in vitro control. %: numbers represent counts; *: significant findings at p < 0.05; **: similar letters in the same column do not differ from each other at a 5% probability level (Kruskal-Wallis test); a, b: differ from each other; a, ab, b: do not differ from each other.

Concentration (%)	Extracts				
	Rosemary	Rue	Citronella	Lemons	Neem
0.0	1.1×10^6 a**^	1.0×10^6 a^	1.0×10^6 a^	1.0×10^6 a^	1.0×10^6 a^
0.4	9.0×10^4 ab^	1.5×10^5 ab^	1.8×10^4 ab^	9.5×10^4 ab^	9.0×10^5 ab^
0.8	8.2×10^4 ab^	1.0×10^4 ab^	5.0×10^2 ab^	9.2×10^3 ab^	8.9×10^4 ab^
1.7	5.3×10^3 ab^	2.0×10^3 ab^	3.1×10^2 ab^	1.0×10^2 ab^	8.7×10^3 ab^
3.2	7.5×10^2 ab^	7.1×10^2 ab^	1.5×10^1 ab^	5.1×10^2 ab^	4.0×10^3 ab^
6.25	1.0×10^2 ab^	5.0×10^1 ab^	0.0 ^b^	9.0×10^1 ab^	5.7×10^2 ab^
12.5	5.2×10^1 ab^	1.7×10^1 ab^	0.0 ^b^	0.8×10^1 ab^	9.0×10^1 ab^
25	0.4×10^1 ab^	0.0 ^b^	0.0 ^b^	0.0 ^b^	6.2×10^1 ab^
50	0.0 ^b^	0.0 ^b^	0.0 ^b^	0.0 ^b^	0.5×10^1 ab^
100	0.0 ^b^	0.0 ^b^	0.0 ^b^	0.0 ^b^	0.0 ^b^
P-value*	0.001	0.001	0.001	0.001	0.001

**Figure 3:**
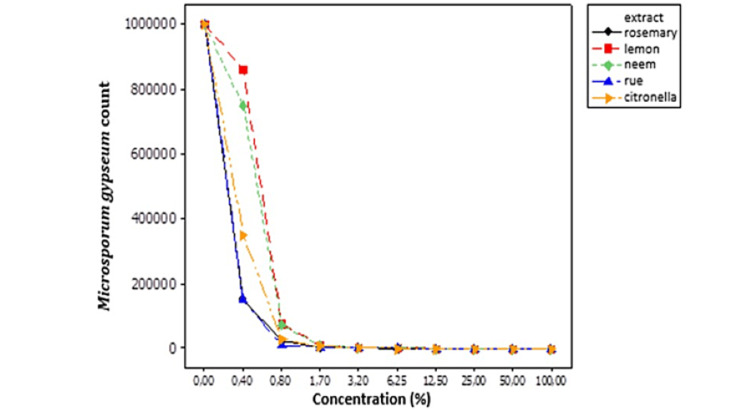
The behavior of Microsporum gypseum in response to different hydroalcoholic extract concentrations of medicinal plants. %: numbers represent counts.

To inhibit *M. gypseum*, high extract concentrations of all extracts were required (Table 4 and Figure 3). Rosemary, rue, citronella, and lemon extracts were efficient at 50%, while neem was efficient at 100%.

**Table 4:** Antimicrobial activity of hydroalcoholic extracts of medicinal plants in the in vitro Microsporum gypseum control. %: numbers represent counts; *: significant findings at p < 0.05; **: similar letters in the same column do not differ from each other at a 5% probability level (Kruskal-Wallis test); a, b: differ from each other; a, ab, b: do not differ from each other.

Concentration (%)	Extracts				
	Rosemary	Rue	Citronella	Lemon	Neem
0.0	1.1×10^6 a**^	1.0×10^6 a^	1.0×10^6 a^	1.0×10^6 a^	1.0×10^6 a^
0.4	1.5×10^5 ab^	1.5×10^5 ab^	3.5×10^5 ab^	8.6×10^5 ab^	7.5×10^5 ab^
0.8	2.3×10^4 ab^	1.0×10^4 ab^	2.9×10^4 ab^	7.5×10^4 ab^	7.0×10^4 ab^
1.7	2.3×10^3 ab^	2.0×10^3 ab^	5.0×10^3 ab^	9.2×10^3 ab^	8.1×10^3 ab^
3.2	2.5×10^2 ab^	7.1×10^2 ab^	5.0×10^2 ab^	1.0×10^3 ab^	2.0×10^3 ab^
6.25	8.0×10^1 ab^	2.5×10^2 ab^	1.6×10^2 ab^	6.1×10^2 ab^	4.4×10^2 ab^
12.5	5.8×10^1 ab^	1.7×10^1 ab^	4.0×10^1 ab^	9.0×10^1 ab^	1.0×10^2 ab^
25	0.3×10^1 ab^	0.2×10^1 ab^	0.2×10^1 ab^	0.1×10^1 ab^	5.0×10^1 ab^
50	0.0 ^b^	0.0 ^b^	0.0 ^b^	0.0 ^b^	0.3×10^1 ab^
100	0.0 ^b^	0.0 ^b^	0.0 ^b^	0.0 ^b^	0.0 ^b^
P-value*	0.001	0.001	0.001	0.001	0.001

**Figure 4:**
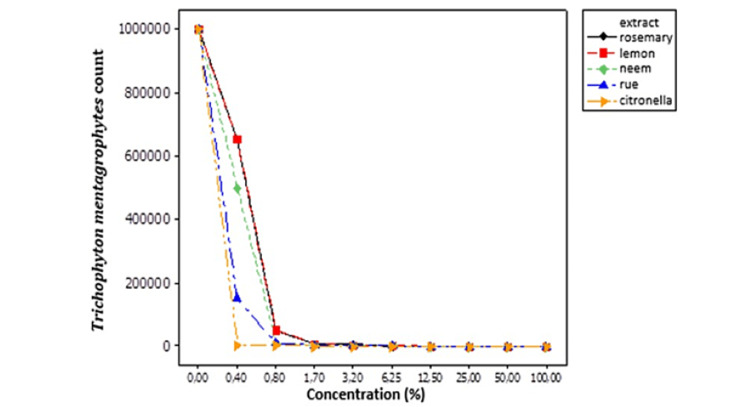
Behavior of Trichophyton mentagrophytes in response to different concentrations of hydroalcoholic extracts of medicinal plants. %: Numbers represent counts

The efficiency of plant extracts in controlling *T. mentagrophytes* is described in Table 5 and Figure 4. Based on the results obtained, rosemary, rue, and neem extracts were effective at 50% concentrations, while lemon was effective at 100%. Greater efficiency was observed when the citronella extract was used because the control concentration was 6.25%.

**Table 5:** Microbial count medians of Trichophyton mentagrophytes for each extract. %: numbers represent counts; *: significant findings at p < 0.05; **: similar letters in the same column do not differ from each other at a 5% probability level (Kruskal-Wallis test); a, b: differ from each other; a, ab, b: do not differ from each other.

Concentration (%)	Extracts				
	Rosemary	Rue	Citronella	Lemon	Neem
0.0	1.1×10^6 a**^	1.0×10^6 a^	1.0×10^6 a^	1.0×10^6 a^	1.0×10^6 a^
0.4	6.5×10^5 ab^	1.5×10^5 ab^	3.3×10^3 ab^	6.5×10^5 ab^	5.0×10^5 ab^
0.8	4.9×10^4 ab^	1.0×10^4 ab^	5.0×10^2 ab^	4.9×10^4 ab^	1.0×10^4 ab^
1.7	7.2×10^3 ab^	2.0×10^3 ab^	1.5×10^2 ab^	7.2×10^3 ab^	2.5×10^3 ab^
3.2	4.2×10^3 ab^	7.1×10^2 ab^	0.4×10^1 ab^	2.0×10^3 ab^	5.5×10^2 ab^
6.25	1.3×10^2 ab^	2.5×10^2 ab^	0.0 ^b^	4.4×10^2 ab^	2.0×10^2 ab^
12.5	2.0×10^1 ab^	1.7×10^1 ab^	0.0 ^b^	1.0×10^2 ab^	1.9×10^1 ab^
25	0.2×10^1 ab^	0.2×10^1 ab^	0.0 ^b^	5.0×10^1 ab^	0.1×10^1 ab^
50	0.0 ^b^	0.0 ^b^	0.0 ^b^	0.3×10^1 ab^	0.0 ^b^
100	0.0 ^b^	0.0 ^b^	0.0 ^b^	0.0 ^b^	0.0 ^b^
P-value*	0.001	0.001	0.001	0.001	0.001

**Figure 5:**
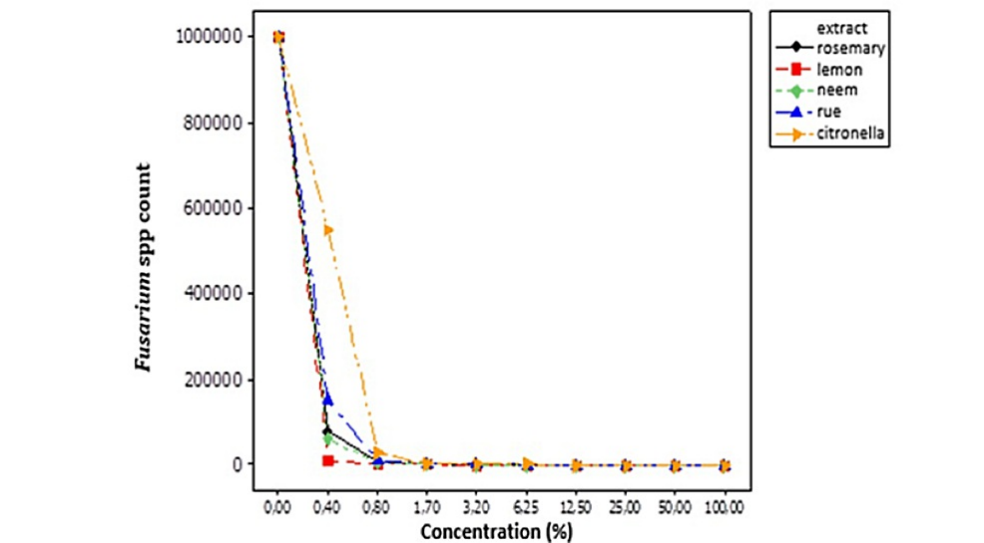
Behavior of Fusarium spp. in response to different concentrations of hydroalcoholic extracts of medicinal plants. %: numbers represent counts.

Table 6 and Figure 5 show the results for the efficacy of different extracts against *Fusarium* spp. There was a higher efficacy of lemon extracts (6.25%), rosemary and neem extracts (12.5%), and rue extracts with the lowest efficiency (50%), although they controlled the fungal species at high concentrations.

**Table 6:** Antimicrobial activity of hydroalcoholic extracts of medicinal plants in the Fusarium spp. in vitro control. %: numbers represent counts; *: significant findings at p < 0.05; **: similar letters in the same column do not differ from each other at a 5% probability level (Kruskal-Wallis test); a, b: differ from each other; a, ab, b: do not differ from each other.

Concentration (%)	Extracts				
	Rosemary	Rue	Citronella	Lemon	Neem
0.0	1.1×10^6 a**^	1.0×10^6 a^	1.0×10^6 a^	1.0×10^6 a^	1.0×10^6 a^
0.4	8.0×10^4 ab^	1.5×10^5 ab^	5.5×10^5 ab^	9.8×10^3 ab^	5.9×10^4 ab^
0.8	8.0×10^3 ab^	1.0×10^4 ab^	2.9×10^4 ab^	5.7×10^2 ab^	4.5×10^3 ab^
1.7	3.0×10^2 ab^	2.0×10^3 ab^	2.5×10^3 ab^	2.9×10^2 ab^	5.0×10^2 ab^
3.2	1.5×10^2 ab^	6.7×10^2 ab^	4.5×10^2 ab^	1.8×10^1 ab^	6.0×10^1 ab^
6.25	0.5×10^1 ab^	5.0×10^1 ab^	2.0×10^2 ab^	0.0 ^b^	0.6×10^1 ab^
12.5	0.0 ^b^	1.7×10^1 ab^	2.7×10^1 ab^	0.0 ^b^	0.0 ^b^
25	0.0 ^b^	0.1×10^1 ab^	0.1×10^1 ab^	0.0 ^b^	0.0 ^b^
50	0.0 ^b^	0.0 ^b^	0.0 ^b^	0.0 ^b^	0.0 ^b^
100	0.0 ^b^	0.0 ^b^	0.0 ^b^	0.0 ^b^	0.0 ^b^
P-value*	0.001	0.001	0.001	0.001	0.001

## Discussion

Fungal infections are characterized by their prevalence, high incidence, treatment difficulty, and morbidity and represent an unsolved major global public health problem [15,26].

The drugs commonly prescribed for fungal infections, especially systemic medications, are known to have many side effects. Moreover, resistant strains of fungi are emerging. The emergence and spread of antimicrobial resistance, as well as the evolution of new strains of disease-causing agents, pose a significant concern for the global healthcare community. Effective disease treatment entails the development of new drugs or potential new drug sources [14]. According to the World Health Organization, medicinal plants can be the best resource for developing a range of drugs [27]. Due to their antimicrobial properties, many plants have been used because of phytochemicals synthesized in the secondary plant metabolism [4,7-9,28-31]. Our study verified that the hydroalcoholic extracts from rosemary, rue, citronella, lemon, and neem exhibited antifungal activity against *C. albicans*, *Fusarium* spp., *A. niger*, and the dermatophytes *M. gypseum* and *T. mentagrophytes* (Table 1).

The neem plant (*Azadirachta indica*) exhibits various medicinal properties. The neem leaf and its constituents have been found to possess immunomodulatory, anti-inflammatory, antimalarial, antifungal, antibacterial, antioxidant, antimutagenic, and anticarcinogenic properties [4,32,33]. Neem leaf and seed extracts have been assessed for antidermatophytic activity. They have been shown to be effective against some dermatophytes such as *Trichophyton rubrum*, *Trichophyton violaceum*, *Microsporum nanum*, *Epidermophyton floccosum*, and the yeast *C. albicans* [3]. These findings corroborate those presented in this study, in which the extract of neem leaves was found to be effective against the dermatophytes *M. gypseum* and *T. mentagrophytes* (Tables 4, 5; Figures 3, 4).

According to Mahmoud et al., testing with aqueous and organic neem leaf extracts showed an inhibitory effect at all concentrations utilized against human pathogenic fungi [4]. These pathogens included four *Aspergillus* species (*A. niger*, *A. flavus*, *A. terrues*, and *A. fumigatus*) known to cause aspergillosis, as well as *M. gypseum* and *C. albicans*, causal agents of dermatophytosis and candidiasis, respectively. All aqueous extract concentrations effectively suppressed the mycelial fungal growth, and the maximum effect was achieved at the highest concentration (20%). This study also observed antifungal activity, but the concentrations ranged from 12.5% to 100% (Table 1).

According to Ali et al., the phytochemical constituents of citrus plants, such as tannins, alkaloids, flavonoids, phenolic compounds, and various other aromatic compounds, are secondary metabolites that can be used to combat microorganisms [17]. The practical exposure and the explanation of the antimicrobial activity on Gram-positive and Gram-negative bacteria and various fungal strains can indicate the broad-spectrum antibiotic compounds present in the extracts [5]. Table 1 shows how the lemon extract exhibited antifungal activity against all strains evaluated. However, it was more efficient for *Fusarium* spp.

Salvadori et al. demonstrated that rue leaf extract has mycelial growth inhibitory capacity against *Colletotrichum gloeosporioides* at 25% when added to a PDA [34].

Costa et al. assessed the composition and antimicrobial activity of the essential oil extracted from fresh citronella leaves [35]. Due to their clinical importance, the strains studied were *Staphylococcus aureus*, *Pseudomonas aeruginosa*, *Escherichia coli*, and *C. albicans*. The results revealed geraniol and citronella as significant constituents and exhibited antimicrobial activity on the tested microorganisms. The authors concluded that citronella oil has the potential to be used as a raw material in pharmaceutical products as well as as a sanitizer in cleaning solutions. Hence, the citronella extract used in this study was more efficient on *A. niger* and *T. mentagrophytes*, although it exhibited antifungal activity against the other strains evaluated (Table 1).

According to Sepahvand et al., in 2016 and 2018, medicinal plants are valuable resources of effective antifungal and therapeutic agents that can be useful in the treatment of various diseases, including fungal infections [26,36]. Such plants are extensively used in traditional and complementary medicine, in unprocessed form, and in modern medicine, either in the processed or purified active substance form. However, more research is needed to develop new drugs from these valuable sources. The lack of bioactive component identification of these extracts was a limitation of this study. Moreover, we did not utilize other plant parts.

## Conclusions

Rosemary, citronella, rue, lemon, and neem hydroalcoholic extracts showed antifungal activity. Rue extracts were more efficient against *C. albicans* and *M. gypseum*. In contrast, the citronella extract inhibited *A. niger* and *T. mentagrophytes*, lemon inhibited *Fusarium* spp., and neem extract inhibited *C. albicans* and *Fusarium* spp. As shown in the present study, the extracts exhibited antifungal properties by eliminating *M. gypseum* at 50% concentration. Therefore, we suggest that these hydroalcoholic extracts can be used for disinfecting the toilets and nursery of the evaluated daycare center.
